# Entheseal Involvement in Spondyloarthritis (SpA) and Gout: An Ultrasound Comparative Study

**DOI:** 10.3389/fmed.2022.871760

**Published:** 2022-05-24

**Authors:** Lucio Ventura-Ríos, Tomas Cazenave, Cristina Hernández-Díaz, Selma Gallegos-Nava, Citlallyc Gómez-Ruiz, Marcos Rosemffet, Karina Silva-Luna, Pedro Rodríguez-Henríquez, Janitzia Vázquez-Mellado, Julio Casasola-Vargas, Esteban Cruz-Arenas, Eugenio M. de Miguel

**Affiliations:** ^1^Laboratorio de Ultrasonido Musculoesquelético y Articular, Instituto Nacional de Rehabilitación Luis Guillermo Ibarra Ibarra, Mexico City, Mexico; ^2^Department of Rheumatology, Instituto de Rehabilitación Psicofísica, Buenos Aires, Argentina; ^3^Department of Rheumatology, Hospital General Dr. Darío Fernández Fierro, Mexico City, Mexico; ^4^Department of Rheumatology, Hospital General de México, Mexico City, Mexico; ^5^Service of Rheumatology, Hospital Universitario “Dr. JoséEleuterio González”, Monterrey, Mexico; ^6^Service of Rheumatology, Hospital General Dr. Manuel Gea González, México City, Mexico; ^7^Instituto Nacional de Rehabilitación Luis Guillermo Ibarra Ibarra, Unidad de Vigilancia Epidemiológica Hospitalaria-Investigación Sociomédica, México City, Mexico; ^8^Rheumatology Service, Hospital Universitario La Paz, Madrid, Spain

**Keywords:** ultrasound, entheses, spondyloarthritis, gout, MASEI

## Abstract

**Objective:**

To compare the assessment of entheses in subjects with spondyloarthritis (SpA) with patients with gout by the Madrid Sonographic Enthesis Index (MASEI).

**Method:**

This cross-sectional study includes videos of entheses evaluated by ultrasound (US) of 30 patients with SpA diagnosed according to the ASAS criteria and 30 patients with gout established by the presence of monosodium urate crystals. Entheses were evaluated for MASEI in 2 Institutes located in two different countries. Demographic and clinical data were registered. Total MASEI score, MASEI-inflammatory, and MASEI-chronic damage were analyzed. Comparisons between groups were obtained by chi-square test and Student's *t*-test. An inter-reading US reliability was realized.

**Results:**

Patients with gout were older and had significantly more comorbidities than those with SpA. The total MASEI score was not significantly different among diseases (*p* = 0.07). MASEI-inflammatory was significantly more prevalent at the Achilles tendon in SpA, while the proximal patellar tendon was in gout. Power Doppler was higher in SpA compared to gout (*p* = 0.005). MASEI-chronic damage related to calcification/enthesophytes predominated in gout (*p* = 0.043), while calcaneal erosions did in SpA (*p* = 0.008). The inter-reader concordance was excellent (0.93, CI 95% 0.87–0.96, *p* = 0.001).

**Conclusions:**

SpA and gout similarly involve entheses according to MASE, however, some inflammatory and chronic lesions differ significantly depending on the underlying disease and tendon scanned.

## Introduction

In patients with spondyloarthritis (SpA), enthesitis is one of the cornerstones of the etiopathogenesis of the disease (1). In axial SpA, the prevalence of peripheral enthesitis is around 25–58% (2). This manifestation is traditionally evaluated by clinical examination based on the presence of pain and/or swelling. However, neither the clinical examination's reliability nor accuracy is satisfactory enough (3–6). In this sense, ultrasound (US) has proven to be a promising imaging technique, since it allows the direct visualization of entheses and related structures (3–7), and it has been observed that it is very sensitive for the evaluation of morpho-structural alterations and changes in blood flow at the entheseal level. In SpA patients, the involvement of the entheses evaluated by the US has been found in up to 98% of cases, with the entheses of the lower limbs being the most frequently affected (3). The reliability of US enthesitis in patients with SpA using OMERACT definitions has been tested in a few studies (8–12).

On the other hand, gout is another inflammatory disease that also affects the entheses (13–15). Several studies have demonstrated the ability of US to differentiate it from other microcrystalline arthropathies in joints (16–18). However, little has been studied about the discriminant capacity of the US at the entheses level (19). In one study that evaluated the discriminant validity of US in SpA, rheumatoid arthritis, gout, chondrocalcinosis, and osteoarthritis in the Achilles tendon, the US shows a potential ability to differentiate between SpA and the other diseases, except for gout (19). So far, the ability of the US to discriminate between SpA and gout has not been evaluated through the identification of elemental lesions of each of these pathologies with MASEI. This index is the most complete and used scoring system, and it has been proven to be reliable and valid for the study of enthesis in diseases other than SpA (20). Although we know that each of these diseases has established diagnostic criteria, the objective of this study was to know if the MASEI in SpA is different compared to gout.

## Materials and Methods

### Study Design

This is a cross-sectional and observational study conducted in México and Argentina. Videos of consecutive patients who were sent to realize MASEI from two rheumatology outpatient clinics to ultrasound units at the National Rehabilitation Institute in México and one rheumatology clinic to the Institute of Psychophysical Rehabilitation in Argentina. The study was approved by the local ethical committee, approval number 10/17, and conducted according to the Declaration of Helsinki. All participants gave written informed consent before realizing the US evaluation.

### Inclusion and Exclusion Criteria

We included videos of 30 consecutive patients with axial or peripheral SpA according to the Assessment of Spondyloarthritis International Society (ASAS) classification criteria were included. Also, videos of 30 patients with gout with diagnosis established by the presence of monosodium urate crystals in synovial fluid or tophus were assessed. The diagnosis of all patients was established by the physician who referred the patients to the ultrasound units. All patients with gout were in an inter-critical period clinically. Patients with gout and psoriasis or inflammatory bowel disease were excluded. Patients who had received oral or injected corticosteroids within 4 weeks before inclusion in the study were also excluded. Demographic data such as age, gender, disease evolution time, comorbidities, and current treatment were recorded. BASDAI, BASFI, BASMI, and MASES were assessed in the case of SpA patients. Entheseal involvement was not an inclusion criterion in none of the diseases.

### Ultrasound Assessment

The videos were obtained and recorded by 2 rheumatologist ultrasonographers (both with more than 10 years of experience), one in each ultrasound unit. The ultrasonographers were blinded to the clinical characteristics of the patients. The videos were recorded. Once all the videos were recorded, they were evaluated by the 5 readers. Triceps brachial, quadriceps tendon, proximal and distal patellar tendon, Achilles tendon, and proximal plantar fascia insertions were evaluated bilaterally, and each enthesis was scanned in longitudinal and transverse planes. The triceps enthesis was examined with the elbow flexed at 90°. Knee entheses evaluation was performed with the patient in supine position and knee flexed at 70° for grayscale and extended for power Doppler (PD). Achilles tendon and plantar fascia were evaluated with the patient in a prone position and their foot flexed at 90°. The US evaluation was blinded and realized independently of the pathology. We used two Esaote MyLab 70^®^ equipment with a 7.5–12 MHz multifrequency linear probe. The vascularization was assessed using PD adjusted with a PRF of 500 Hz and gain from 50 to 55 dB.

According to MASEI, the following lesions were evaluated: pathologic structural change and thickening of the tendon at the site of insertion, calcification/enthesophyte, bursitis, bone erosion, and PD signal. The pathologic structural change was defined as a loss of a fibrillar pattern, hypoechoic appearance, or fusiform thickening; the following criteria were used for abnormal structure thickness: quadriceps tendon thickness >6.1 mm, proximal and distal patellar tendon >4.0 mm, Achilles tendon >5.29 mm, and plantar fascia >4.4 mm. Bone erosion was defined as cortical breakage with bone contour defects in 2 perpendicular planes. Calcification/enthesophyte was scored 0 if absent, 1 for small calcification or ossification with an irregularity of cortical bone, 2 if a clear presence of enthesophytes (hyperechoic spurs forming at a tendon insertion into the bone, growing in the direction of the natural pull of the tendon involved), or if medium-sized calcifications or ossification were seen and 3 for large calcifications or ossifications (21). According to OMERACT, merging some components like calcifications and enthesophytes is an adequate strategy to improve reliability, because sometimes they have the same appearance (22). Bursitis was defined as a well-circumscribed, localized anechoic or hypoechoic area at the site of an anatomical bursa, which was compressible by the transducer (21). The presence of PD signal was considered when seen at bone insertion (<2 mm from the cortical bone), different from reflecting surface artifact or nutrition vessel signal, with or without cortical irregularities, erosions, or enthesophytes, according to OMERACT definition (22). The MASEI score was categorized in inflammatory lesions (thickening structural changes, bursitis, and vascularization) and chronic damage (calcifications/enthesophytes and bone erosions). The inter-reader agreement of total MASEI scores was performed among 5 ultrasonographers and the expert in the MASEI index (de Miguel E) online.

### Statistical Analysis

Continuous data are described as the mean and standard deviation and categorical variables were expressed as frequencies and percentages. The normality of the continuous variables was probed by the Shapiro-Wilk test. The chi-square distribution was applied to compare categorical variables between groups. The student's *t*-test was used to contrast the total MASEI scores between groups. To analyze inter-reader agreement for continuous data we used intraclass correlation coefficient (ICC) with a 95% confidence interval. A *p* < 0.05 was considered statistically significant. Statistical analysis was performed in SPSS for Windows version 22.

## Results

Clinical and demographic characteristics are shown in Table 1. The average age of patients and prevalence of comorbidities were significantly higher in the gout group than in the SpA. There was no significant difference in disease duration, weight, height, and body mass index (BMI) between groups. As expected, uric acid levels were significantly higher in patients with gout than with SpA. A high percentage had tophaceous gout. All patients with gout were receiving hypouricemic treatment; most of them had allopurinol. The SpA group received 43% methotrexate, 15% sulfasalazine, and 23% anti-TNF. A similar percentage of patients in both groups used non-steroidal anti-inflammatory drugs. The average BASDAI score was high suggesting SpA activity. BASFI and BASMI showed high dysfunction and decrease in spinal mobility, respectively. The median of MASES was 4.

**Table 1:** Clinical and demographics characteristics among groups.

	**Gout**	**SpA**	***p***
	***n* = 30**	***N* = 30**	
Age years ± SD	54.1 (11.1)	45.7 (11.6)	0.005
Sex Men (%)	26 (86.7%)	21 (70.0%)	0.559
Women (%)	4 (13.3%)	9 (30.0%)	0.267
Disease duration years ± SD	10.2 (2.9)	9.7 (7.2)	0.324
Patients with comorbidities number (%)	25 (83.3%)	7 (23.3%)	0.002
Weight kg mean ± SD	75.7 + 9.5	70.6 ± 11.2	0.062
Height m mean ± SD	1.63 +1.6	1.64 ± 0.7	0.906
BMI mean ± SD	28.5 + 5.3	26.1 ± 4.3	0.059
Uric Acid level mg/dL mean ± SD	7.6 ± 1.7	5.5 ± 1.4	0.001
SpA axial / peripheral number (%)	NA	13 (43)/17 (56)	
Tophaceous gout *n* (%)	19 (63.3%)	NA	
**Treatment**			
Allopurinol	76%	NA	
Febuxostat	24%	NA	
Colchicine	53%	NA	
Non-steroidal anti-inflammatory drugs	48%	49%	
Methotrexate	NA	43%	
Sulphasalazin	NA	15%	
Anti-TNF	NA	23%	
BASDAI	NA	5.6 ± 3.8	
BASFI	NA	7.9 ± 3.9	
BASMI	NA	3.6 ± 1.2	
MASES median (min-max)	NA	4 (0–8)	

*NA. Not Applicable*.

**Figure 1:**
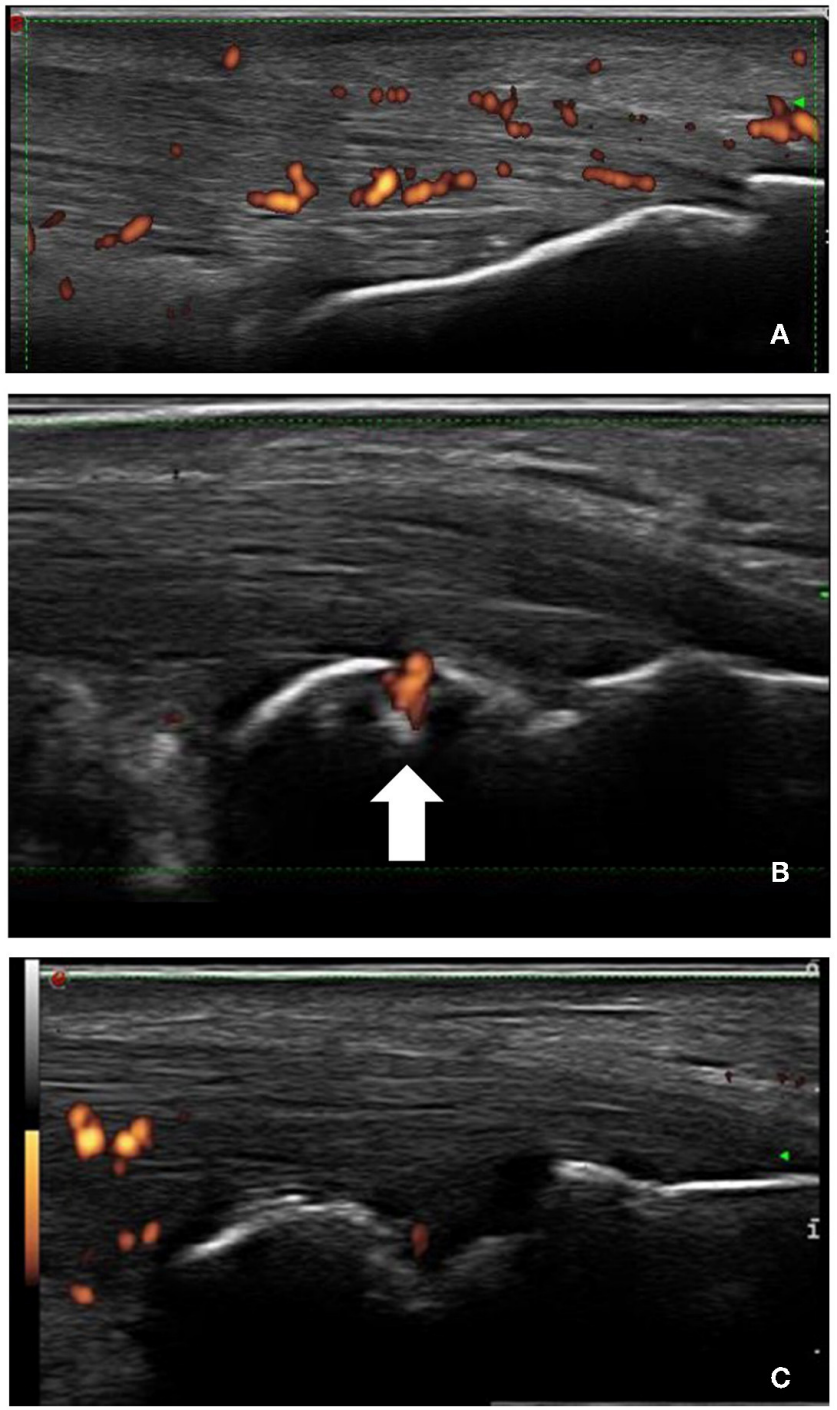
Longitudinal scans of Achilles tendon in SpA. **(A)** Increased thickness of tendon insertion, enthesophyte (the step up of the bony prominence at the end of the normal bone contour), and power Doppler signal. **(B)** The arrow shows PD signal inside bone erosion and **(C)** arrowhead shows retrocalcaneal bursitis.

**Figure 2:**
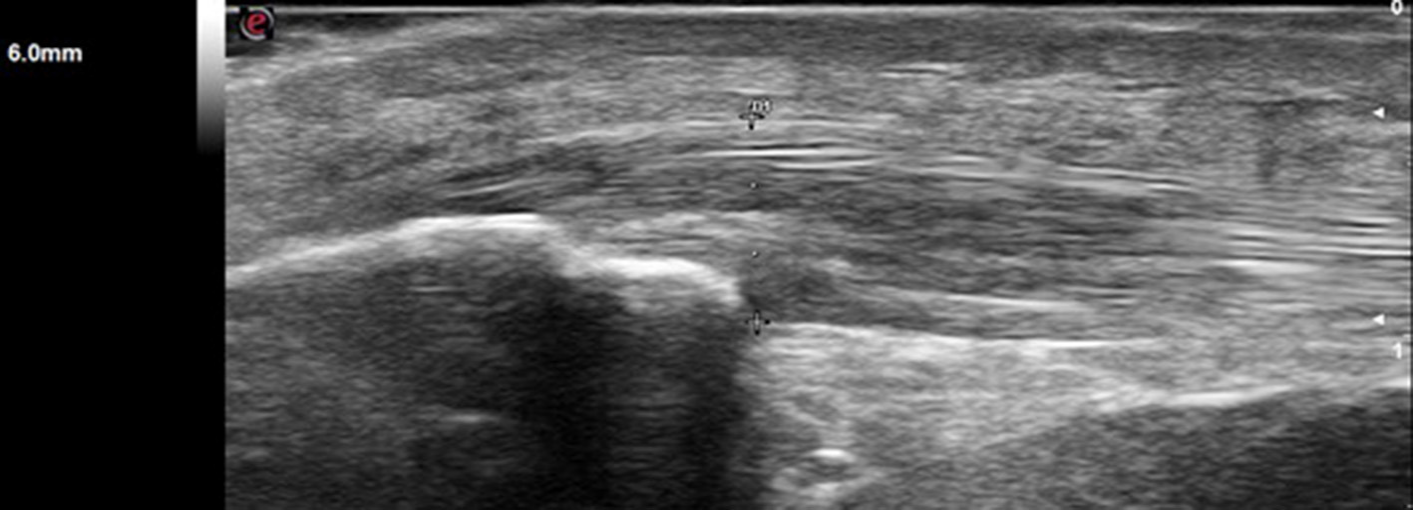
Longitudinal view of proximal patellar tendon shows increased thickness (> 4.0 mm) and hypoechogenicity in a patient with gout.

**Figure 3:**
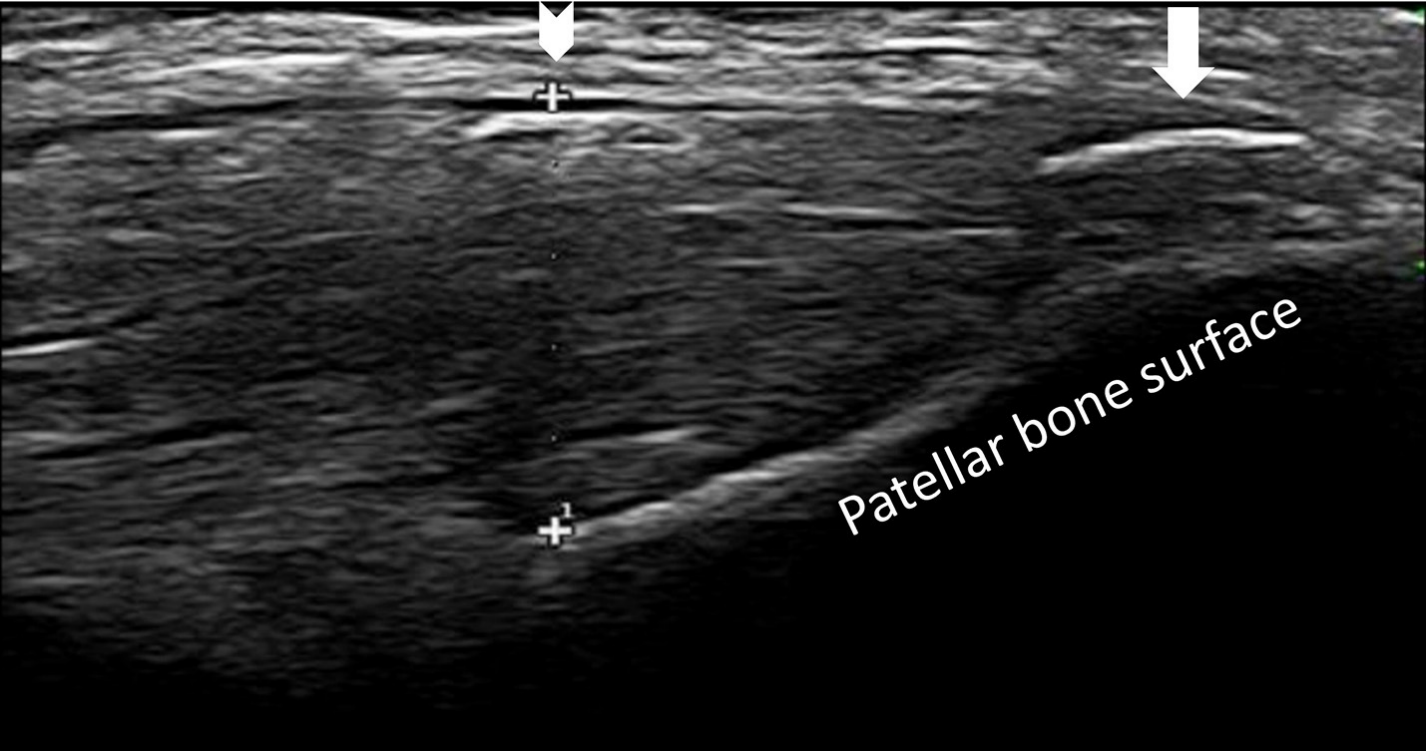
Longitudinal scan of the distal patellar tendon in a patient with gout shows increased thickness, hypoechogenicity (arrowhead), and calcification (arrow).

Table 2 shows differences between groups related to lesions in the entheses evaluated. The site that was most frequently affected with structural change, thickness, erosion, bursitis, and PD signal was the Achilles tendon in patients with SpA (Figures 1A–C). In contrast with gout where the presence of structural change and thickening in the proximal patellar tendon was higher (Figure 2). The presence of small enthesophytes in the quadriceps tendon was significantly greater in gout than in SpA (Figure 3), whereas pathologic structural changes of the same tendon prevailed in gout. There was no significant difference in the distal patellar tendon, triceps tendon, and plantar fascia between groups.

**Table 2:** Comparison of US findings of entheses between groups.

**Enthesis**	**Gout** **30 patients** **60 entheses** ***n* (%)**	**Spondyloarthritis** **30 patients** **60 entheses** ***n* (%)**	***P***
**Quadriceps tendon**			
Structural change	9 (15.0)	19 (31.6)	0.089
Thickening	8 (13.3)	13 (21.6)	0.382
Erosion	0 (0)	1 (1.6)	0.321
Calcification/enthesophyte (grade)			
0	35 (58.3)	40 (66.6)	0.644
1	19 (31.6)	5 (8.3)	**0.008**
2	6 (10.0)	9 (15.0)	0.605
3	0 (0)	6 (10.0)	0.130
Power Doppler signal	0 (0)	0 (0)	-
**Proximal Patellar tendon**			
Structural change	24 (40.0)	8 (13.3)	**0.008**
Thickening	21 (35.0)	9 (15.0)	**0.044**
Erosion	2 (3.3)	3 (3.3)	0.998
Calcification/enthesophyte			
0	39 (65.0)	45 (75.0)	0.585
1	19 (31.6)	10 (16.6)	0.137
2	2 (3.3)	2 (3.3)	1.000
3	0 (0.0)	1 (1.6)	0.321
Power Doppler signal	0 (0.0)	6 (10.0)	0.130
**Distal patellar tendon**			
Structural change	24 (40.0)	22 (36.6)	0.882
Thickening	21 (35.0)	22 (36.6)	0.998
Erosion	2 (3.3)	1 (1.6)	0.998
Calcification/enthesophyte			
0	42 (70.0)	49 (81.6)	0.529
1	14 (23.3)	7 (11.6)	0.190
2	4 (6.6)	2 (3.3)	0.683
3	0 (0.0)	0 (0.0)	-
Power Doppler signal	2 (3.3)	5 (8.3)	0.449
Bursitis infrapatelar	8 (13.3)	10 (16.6)	0.813
**Achilles tendon**			
Structural change	7 (11.6)	18 (30.0)	**0.045**
Thickening	4 (6.6)	14 (23.3)	**0.033**
Erosion	0 (0)	12 (20.0)	**0.005**
Calcification/enthesophyte			
0	39 (65.0)	35 (58.3)	0.727
1	13 (21.6)	5 (8.3)	0.099
2	6 (10.0)	16 (26.6)	0.055
3	2 (3.3)	3 (5.0)	0.998
Power Doppler signal	0 (0.0)	8 (13.3)	**0.045**
Retrocalcaneal bursa	0 (0.0)	9 (15.0)	**0.029**
**Plantar fascia**			
Structural change	20 (33.3)	14 (20.0)	0.391
Thickening	20 (33.3)	14 (20.0)	0.391
Erosion	2 (3.3)	2 (3.3)	1.000
Calcification/enthesophyte			
0	39 (65.0)	39 (65.0)	1.000
1	15 (25.0)	10 (16.6)	0.423
2	6 (10.0)	9 (15.0)	0.605
3	0 (0)	0 (0.0)	-
Power Doppler signal	0 (0)	1 (1.3)	0.321
**Triceps tendon**			
Structural change	14 (23.3)	9 (15.0)	0.404
Thickening	14 (23.3)	9 (15.0)	0.404
Erosion	9 (15.0)	12 (20.0)	0.662
Calcification/enthesophyte			
0	40 (66.6)	48 (80.0)	0.455
1	10 (16.6)	5 (8.3)	0.301
2	10 (16.6)	5 (8.3)	0.301
3	0 (0)	0 (0)	-
Power Doppler signal	0 (0.0)	1 (1.3)	0.321

*The bold values indicate the values that were significantly different between groups*.

Table 3 presents data related to MASEI. The total MASEI scores were higher in patients with SpA than gout, however, it was not significant. Regarding MASEI-inflammatory there were no differences in structural changes and thickening among groups, but the PD signal was significantly higher in SpA than gout. In respect of MASEI-chronic damage, the gout group had significantly higher calcifications/enthesophytes, nonetheless, bone erosions prevailed in the SpA group.

**Table 3:** MASEI score in 360 entheses in gout compared with SpA.

**Total MASEI score means ±DS**	26.0 ± 8.0	34.5 ± 14.8	***p*** 0.07
**MASEI-inflammatory**	*n* (%)	*n* (%)	
Structural change	99 (27.5%)	90 (25.0%)	0.560
Thickening	88 (24.4%)	81 (22.5%)	0.644
Power Doppler signal	2 (0.5%)	21 (5.8%)	**0.002**
Bursitis			
**MASEI-chronic damage**	8 (2.2%)	19 (5.2%)	0.054
Erosion	15 (4.1%)	41 (11.3%)	**0.008**
Calcification/enthesophyte	126 (35.0%)	95 (26.3%)	**0.043**

*The bold values indicate the values that were significantly different between groups*.

Inter-reader agreement for total MASEI score was excellent (0.93, 95% CI, 0.87–0.96, *p* = 0.001).

## Discussion

Although the US has proven to be a reliable and valid technique to evaluate enthesitis in SpA (23), little has been analyzed about its discriminant validity. In a study, the power of discrimination of US was evaluated by MASEI between different chronic diseases in the Achilles enthesis, showing that this index lacks validity to discriminate SpA from gout (19). Probably the fact that there is a single enthesis being assessed, limits the possibility of establishing the discriminant validity of an imaging technique. Other entheses different from the Achilles tendon are affected in gout, such as patellar tendon and quadriceps as observed in other studies (14, 15, 24). Therefore, in the present study, we decided to evaluate the 6 bilateral entheses included in the MASEI score.

As previously reported (19), we found significantly more structural changes/thickening and bone erosion in the Achilles tendon in SpA than in gout. This enthesis seems to be the most affected in SpA according to what has been reported (7, 25). de Miguel and cols, using MASEI had demonstrated that the presence of PD signal and bone erosion have a better predictive value for inflammatory enthesitis in SpA (21). Besides, as we have observed a higher prevalence of bone erosions in SpA than in gout, another study has found similar results (19). In a systematic review, significantly more erosions in the calcaneal enthesis were observed in SpA compared with the healthy population (26). Additionally, the presence of bursitis at the level of the calcaneal enthesis accompanying the inflammatory lesions can contribute to differentiating patients with SpA (27). By contrast, despite crystal deposition in gout, structural changes are infrequent in the Achilles tendon, as it has been shown in people with tophaceous gout where many of the characteristics observed were not disease-specific (28).

Structural change in the quadriceps tendon tended to be significantly greater in the SpA group than in gout, suggesting that it is one of the sites that could differentiate SpA from healthy subjects or other diseases (25, 29, 30). Moreover, patients with gout had a significantly greater presence of small calcifications/enthesophytes in quadriceps than those with SpA, as a recent study shows, where this tendon was the most involved in both diseases, therefore it is a site that requires more attention (31).

On the other hand, structural change in the proximal patellar tendon was significantly greater in gout than in SpA. In general, the patellar tendon seems to be the most affected in gout in several studies (1, 14, 15, 24). We did not find other significant differences in this tendon and the distal patellar tendon and plantar fascia because they are affected similarly in both diseases (31). Concerning the triceps tendon, there was a trend for a higher percentage of both inflammatory and chronic damage in the gout group, however, there were no significant differences among groups. It has been reported that it is the second tendon most affected in gout, affecting almost 50% of patients while, in SpA it is involved in around 12% of independent studies (14, 32). We consider that this is the first time that these entheses have been compared in these diseases.

Respecting the MASEI-Inflammatory index, we did not find significant differences in structural change and thickness because gout affects entheses as frequently as SpA does (31). Only the presence of PD was significantly present in patients with SpA and mainly observed in calcaneal enthesis (30, 33). The low prevalence of PD in the population with gout contrasts with other studies (28, 31). Factors associated with vascularization are advanced age and high uric acid levels. It has been shown that PD signal significantly decreased at 2 years of urate-lowering therapy (34).

In MASEI-chronic damage, the calcifications/enthesophytes were most frequently demonstrated in gout like the other study (19). It is probable that calcifications are a predominant characteristic associated with the deposit of MSU in entheses, as shown by animal models of enthesitis, where local injection of monosodium urate crystals into the metatarsal entheses of oxidative-burst-deficient (Ncf1^**^) mice developed chronic enthesitis accompanied by massive enthesophytes by resonance magnetic imaging (35). In addition, it has been observed that advanced age and belonging to the male sex are associated with greater structural damage, factors that prevailed in our gout group (20).

The total MASEI score was higher in SpA than gout but there were no significant differences. According to the original study, 18 points would be the best cut-off point to differentiate patients with SpA from controls (21). However, patients with longstanding gout develop a higher frequency of chronic damage, specifically calcifications/enthesophytes in multiple entheses, which increases the index. Therefore, the MASEI would have limitations to be used to differentiate between both groups. It is important to note that most of the patients with gout had the tophaceous variety, which could contribute to having a higher MASEI score (13). Discriminant validity of MASEI has been studied in other diseases like Behcet and Fibromyalgia in which, the entheses are often not affected, giving low scores, thus facilitating discrimination, in contrast, in diseases such as gout, the discrimination by this score can be more difficult (36, 37).

Finally, the inter-reading concordance was excellent. The performance of inter-reader exercise has a great influence to improve reliability and our group has carried out this type of exercise periodically (38, 39). The other study has shown excellent inter-observer agreement for quantitative data (37).

## Limitations and Strengths

One of the main limitations of our study was a relatively low number of patients, however, 720 entheses is a good number to consider. Another limitation was the age difference which was greater in the gout group; a bias that is difficult to correct given that patients with gout start their disease later. This condition probably accounts for a higher frequency of calcifications in the gout group, however, for the analysis of differences between groups we are not exclusively based on this lesion. Another constraint is that all patients with gout were receiving hypouricemic treatment and more than 40% of the SpA group were receiving immunosuppressive therapy and just over 20% biological therapy, conditions that could reduce the presence of PD in the entheses. Another weakness of the study is that we did not record the physical activity of the patients because it has been described those individuals with a greater demand for physical activity develop more structural and inflammatory changes in entheses. Additionally, the comorbidities observed in patients with gout could have contributed to the entheseal condition. All the same, one of the strengths of the study is that it includes a binational multicenter sample and patients were of real life.

## Conclusions

The total MASEI could not discriminate between SpA and gout, however, some inflammatory and chronic lesions differ significantly depending on the underlying disease and tendon explored. In the Achilles tendon, this index shows the ability to differentiate SpA from gout due to having a higher prevalence of structural change, thickness, bursitis, erosions, and PD signal. Gout induces the development of calcifications/enthesophytes which increases the total index. Entheseal involvement in gout is almost as frequent as in SpA, therefore its evaluation is necessary.

## Data Availability Statement

The original contributions presented in the study are included in the article/supplementary material, further inquiries can be directed to the corresponding authors.

## Ethics Statement

The studies involving human participants were reviewed and approved by Research Committee of the National Institute of Rehabilitation. The patients/participants provided their written informed consent to participate in this study.

## Author Contributions

LV-R and EM contributed to study conception, design, data collection, and manuscript drafting. CH-D, TC, SG-N, CG-R, MR, KS-L, PR-H, JV-M, and JC-V participated in data collection and manuscript drafting. EC-A contributed to statistical analysis. All authors read and approved the final manuscript.

## Conflict of Interest

The authors declare that the research was conducted in the absence of any commercial or financial relationships that could be construed as a potential conflict of interest.

## Publisher's Note

All claims expressed in this article are solely those of the authors and do not necessarily represent those of their affiliated organizations, or those of the publisher, the editors and the reviewers. Any product that may be evaluated in this article, or claim that may be made by its manufacturer, is not guaranteed or endorsed by the publisher.
